# Drought-Induced Leaf Proteome Changes in Switchgrass Seedlings

**DOI:** 10.3390/ijms17081251

**Published:** 2016-08-02

**Authors:** Zhujia Ye, Sasikiran Sangireddy, Ikenna Okekeogbu, Suping Zhou, Chih-Li Yu, Dafeng Hui, Kevin J. Howe, Tara Fish, Theodore W. Thannhauser

**Affiliations:** 1 Department of Agricultural Sciences, Tennessee State University, 3500 John Merritt Blvd, Nashville, TN 37209, USA; zye@my.tnstate.edu (Z.Y.); sangisasi@gmail.com (S.S.); iyk_oc@yahoo.com (I.O.); 2 Department of Biological Sciences, Tennessee State University, 3500 John Merritt Blvd, Nashville, TN 37209, USA; cyu@my.tnstate.edu (C.-L.Y.); dhui@tnstate.edu (D.H.); 3 Functional & Comparative Proteomics Center, USDA-ARS, Cornell University, Ithaca, NY 14853, USA; kjh46@cornell.edu (K.J.H.); tlf26@cornell.edu (T.F.)

**Keywords:** physiological properties, isobaric tags for relative and absolute quantitation (iTRAQ), ProteoMiner, functional pathways, abscisic acid (ABA) signaling, “Sandwich” plant growth system

## Abstract

Switchgrass (*Panicum virgatum*) is a perennial crop producing deep roots and thus highly tolerant to soil water deficit conditions. However, seedling establishment in the field is very susceptible to prolonged and periodic drought stress. In this study, a “sandwich” system simulating a gradual water deletion process was developed. Switchgrass seedlings were subjected to a 20-day gradual drought treatment process when soil water tension was increased to 0.05 MPa (moderate drought stress) and leaf physiological properties had expressed significant alteration. Drought-induced changes in leaf proteomes were identified using the isobaric tags for relative and absolute quantitation (iTRAQ) labeling method followed by nano-scale liquid chromatography mass spectrometry (nano-LC-MS/MS) analysis. Additionally, total leaf proteins were processed using a combinatorial library of peptide ligands to enrich for lower abundance proteins. Both total proteins and those enriched samples were analyzed to increase the coverage of the quantitative proteomics analysis. A total of 7006 leaf proteins were identified, and 257 (4% of the leaf proteome) expressed a significant difference (*p* < 0.05, fold change <0.6 or >1.7) from the non-treated control to drought-treated conditions. These proteins are involved in the regulation of transcription and translation, cell division, cell wall modification, phyto-hormone metabolism and signaling transduction pathways, and metabolic pathways of carbohydrates, amino acids, and fatty acids. A scheme of abscisic acid (ABA)-biosynthesis and ABA responsive signal transduction pathway was reconstructed using these drought-induced significant proteins, showing systemic regulation at protein level to deploy the respective mechanism. Results from this study, in addition to revealing molecular responses to drought stress, provide a large number of proteins (candidate genes) that can be employed to improve switchgrass seedling growth and establishment under soil drought conditions (Data are available via ProteomeXchange with identifier PXD004675).

## 1. Introduction

Switchgrass (*Panicum virgatum*), has been selected as a model herbaceous bioenergy species in the USA due to its high biomass yield, strong tolerance to drought and flooding conditions, relatively low herbicide and fertilizer input requirements, and widespread adaptability to temperate climate [1,2,3]. Recently, a shortage of fresh water and increasingly severe drought have become a significant challenge to crop production [4]. Based on data from the National Weather Service Centers for Environmental Prediction [5], soil moisture contents in the topsoil layer have declined over the past decade (2005–2015) in many regions of the USA, especially in central states.

Drought tolerance is one of the most striking physiological properties of switchgrass. Mature plants have a very deep root system and a highly efficient C_4_ metabolic pathway [6]. However, switchgrass plants are slow to establish in the field, often requiring two to three growing seasons to develop deep root systems. During the early stages of growth when seedlings have a relatively shallow root distribution (0–15 cm) in the top soil, these plants are very susceptible to both periodic and long-term drought conditions [7]. A field trial shows that drought significantly affected seedling growth of switchgrass in the first year. Furthermore, biomass yield declined greatly after three consecutive years of drought [8]. Thus, developing switchgrass plants with strong drought tolerance during the early stages of growth is an effective strategy to ensure high biomass yields during subsequent years in the field.

Plant growth depends on cell division, cell enlargement, and differentiation [9]. Under drought conditions, cell elongation and division are both suppressed by the reduced photosynthesis driven by diminished CO_2_ influx and limitation of carboxylation by abscisic acid (ABA)-dependent stomatal closure [9,10,11,12]. On the other hand, stomatal closing has been viewed as a drought tolerance mechanism to avoid excess water loss via transpiration. A set of physiological parameters related to drought tolerance has been identified including leaf relative water content (RWC), electrolyte leakage (EL), photosynthetic rate (Pn), stomatal conductance (*g*_s_), transpiration rate (Tr), intercellular CO_2_ concentration (Ci), and water use efficiency (WUE) [13]. Thus, whether plants can sustain active growth or just survive the water-deficient conditions depends on how efficiently they regulate these complex processes.

Proteins are the primary molecules that carry out various biological functions in cells and in an entire organism [14]. Alterations in proteome composition provide the basis for a plant to perform different biological functions, including adapting to changing and/or suboptimal environmental conditions [15,16,17,18,19,20,21]. With the rapid development of proteomic technologies, two-dimensional liquid chromatography, in combination with multiplexed quantitative techniques such as isobaric tags for relative and absolute quantitation (iTRAQ), provides the ability to perform relative or absolute quantification of proteomes [22,23,24,25,26]. Quantitative proteomics using the shot-gun bottom-up approach has been used to evaluate drought-responsive proteins in important crop species, such as rice, maize, wheat, cotton, amaranth, alfalfa, sugar beets, and tomatoes [18,20,27,28,29,30,31,32,33,34,35,36,37]. Conclusively, these proteomics studies have significantly increased our understanding of molecular regulation at the translational and post-translational levels in plants.

The separation and detection of all proteins contained in any given proteome remains a challenge because the analysis of low-abundance proteins is difficult in the presence of the highly abundant proteins. Characterization of the photosynthetically active leaf proteome is a very difficult task as the ribulose-1,5-biphosphate carboxylase/oxygenase (Rubisco) proteins would account for approximately 40% of total protein content [38]. An earlier study using immunoaffinity subtraction of Rubisco was able to increase the resolution of more protein species in leaf protein samples [39]. However, those antibodies are very expensive, which limits their usage in large quantitative proteomics experiments (unpublished data, Zhou, Tennessee State University, Nashville, TN, USA, 2016).

The ProteoMiner protein depletion/enrichment technology, which employs a large, highly diverse bead-based library of combinatorial peptide ligands, has proven to be a powerful tool for uncovering low-abundance proteins. Using this approach, Fasoli et al. detected 79% more proteins from spinach leaves than could be detected without the depletion/enrichment process [40]. More importantly, the ProteoMiner protein enrichment method produces highly stable and reproducible results, which is extremely important in quantitative proteomics where two or more samples are analyzed in each treatment condition [41,42].

This study was carried out with a goal to understand the changes in leaf proteome in switchgrass under drought stress and to develop the association between the expression of these proteins and the physiological properties that give rise to drought tolerance. As described above, removal of highly abundant Rubisco protein is an effective strategy for increasing the overall number of identified proteins, thus the ProteoMiner depletion/enrichment procedure was performed to reduce the scale of dynamic range in protein abundance. By enabling the identification of low-abundance proteins and increasing the number of proteins quantified, this study provides an in-depth understanding of systemic changes in the drought-induced proteomes in switchgrass seedlings.

## 2. Results

### 2.1. Drought-Induced Physiological Properties of Switchgrass

**Figure 1:**
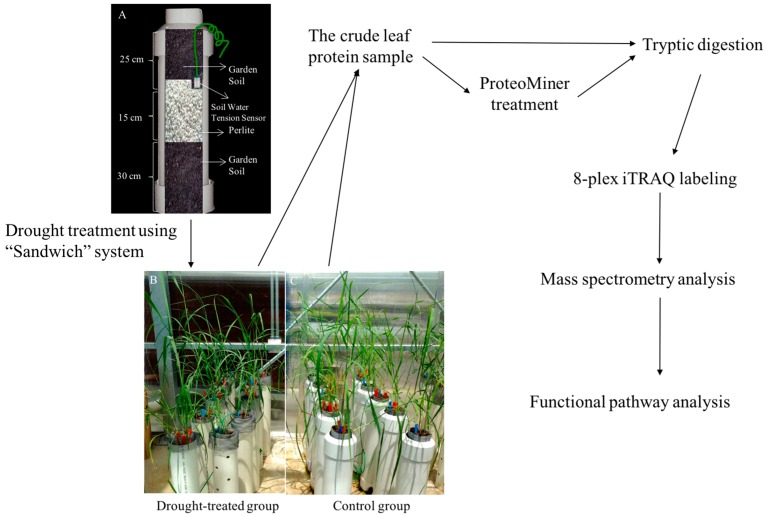
Flow chart of the drought treatments and quantitative proteomics procedure. Plants were grown in a “sandwich” system (**A**); During the 20th day of the water withholding period, physiological data were recorded on both drought-treated (**B**); and well-watered control plants (**C**). Leaf protein samples were extracted followed by the ProteoMiner enrichment. Quantitative proteomics analysis was performed using the crude leaf protein extracts and the ProteoMiner-enriched samples. Functional pathways were developed using information on the drought-induced changes in the leaf proteomes, and the association between protein expression and physiological properties was developed focusing on drought stress tolerance.

Sixteen days after the initiation of water withholding, young leaves on drought-treated plants started to show signs of wilting as the soil water tension of treated groups reached 0.05 MPa. Twenty days after water withholding, soil water tension increased to 0.08 ± 0.02 MPa (Table 1). At this time, the relative growth of drought-treated plants was reduced significantly (a 20% decrease), as well as the stomatal conductance and transpiration rate (*p* < 0.01), compared to the untreated control plants. The water use efficiency, which is defined as the ratio of the photosynthetic rate to the transpiration rate [43], showed an 7.1% increase in the drought-treated group, which was significantly higher than the untreated control plants (*p* < 0.01). Changes in these physiological properties showed that leaves and plants as a whole experienced a progressive drought-stress during the 20 days of withholding water. At this time-point (20 days after withholding water), the drought treatments were terminated and tissues were harvested for further analysis (Figure 1).

### 2.2. Effects of the ProteoMiner Enrichment Process on the Identification of Leaf Proteome

The ProteoMiner enrichment method is used to increase the relative concentration of low-abundance proteins by depleting those high-abundance proteins in a protein sample, and thus to increase the depth of coverage of the leaf proteomes to be identified in a proteomics analysis. In plant leaf proteomes, more than 40% of the total leaf protein content consists of Rubisco [38]. One-dimensional gel electrophoresis showed that in the ProteoMiner-treated (PMT) samples, the band intensity of high abundance proteins (i.e., Rubisco protein) was reduced, whereas the intensity of several weaker protein bands was increased, compared to the counterparts of the Crude Leaf Extracts (CLE) protein samples (Figure S1). Analysis of the peptide numbers for the proteins detected in the PMT sample indicates that 1101 proteins were identified by a greater number of peptides after ProteoMiner enrichment—for instance, the number of peptides in an adenylate kinase protein (Pavir.Fa02159.1) was increased from 29 in CLE to 189 in PMT samples. On the other hand, 1876 proteins were identified by fewer peptides. For example, the number of peptides in Rubisco subunits including Pavir.Cb01593.1, Pavir.Cb01387.1, and Pavir.J32704.1 was decreased (Figure S2, Table S1-1,2). These results demonstrate that ProteoMiner did deplete the concentration of the highly abundant proteins while simultaneously enriching low-abundance proteins.

### 2.3. Identification of Quantified Proteins

In this study, 7006 proteins were identified in the switchgrass leaf proteome with the assistance of the ProteoMiner enrichment method (Table 2, Tables S1-1–3 and S2). A total of 5493 proteins were identified in the CLE samples and 4839 unique proteins were identified in the PMT samples. Between the CLE and PMT samples, 3326 proteins overlapped. The use of a ProteoMiner enrichment step resulted in the identification of 1513 proteins that were not found in the CLE samples. It appears that the ProteoMiner enrichment is complementary to the analysis of the crude leaf protein extracts, and a combination of both approaches was shown to quantify more proteins than either individually.

Among the total identified proteomes, 81.1% of them (5680/7006) contained at least two unique peptides (Table 2). Quantitative analysis revealed that 257 proteins, which was approximately 4% of the total quantified proteomes (257/7006), passed the threshold value of ±2σ (standard deviation), *p* < 0.05 (*t*-test and false discovery rate (FDR) corrections), and fold change <0.6 or >1.7. These proteins were considered significantly changed under the drought treatment conditions. Among the 257 drought-induced significant proteins, 55 proteins showed consistent changes in both the CLE and PMT protein samples, 150 proteins were found only in CLE samples, and 52 proteins were only identified in PMT samples (Tables S1-5 and S3). In addition, the false negative rate (β) was calculated as 0.02 by summing the probabilities that each of the proteins judged to be unchanged was in fact differentially expressed. This suggests that the power of the experiment was very high (*p* = 1 − β = 0.98).

MapMan is a bioinformatics tool for developing the associations between gene (protein) expression and cellular processes, but this offline program only performs analysis of genomes contained in the MapMan Store. As the annotated switchgrass genome database is not listed, the program will not recognize the protein accession identity and therefore cannot map the protein expression data to biological functions. Instead, in this study, the *Arabidopsis* *thaliana* accessions annotated for those drought-induced switchgrass proteins were used when developing the functional pathways (Figure S3). Results showed that each functional group contained upregulated and downregulated proteins. A large number of the significantly changed proteins are associated with RNA transcription/processing, protein synthesis, and protein degradation pathways (Table 3).

### 2.4. Proteins in Regulation of Transcription and Translation

For proteins involved in gene transcription, several members of the G2-like, myeloblastosis (MYB) and bZIP transcription factors (TFs) were identified. A MYB-related transcription factor TRY (Triptychon) (Pavir.Eb02165.1) and a G2-like transcription factor APL (altered phloem development) (Pavir.Fa01260.1) were significantly reduced under drought stress. The former TF did not pass the threshold as a significant protein in CLE, and the latter TF was identified only in the PMT samples. The GBF (G-box binding factor) (Pavir.Ea03718.1), a member of the bZIP TFs family involved in ABA and stress signaling [44], was significantly increased (>2-fold) (Table S1-4), and it was identified in both CLE and PMT samples.

Proteins involved in protein synthesis and degradation were altered. The chloroplast-targeted FtsH protease (Pavir.J13145.1) was up-regulated at a higher than four-fold level. Moreover, the relative abundance of a senescence-specific Cys-protease protein (SAG) (Pavir.J08126.1) markedly declined in response to drought stress. Regarding to changes in protein synthesis, drought stress induced a plastid-specific 50S ribosomal protein (PSRP) (Pavir.Ea00033.1), which is an important member of the translation machinery in chloroplasts. However, the drought-induced significant change was found only in PMT samples, not in CLE samples (Table S1-4).

### 2.5. Cell Division and Cell Wall Modification

Two proteins involved in the cell cycle and cell division were identified. Pavir.Gb00127.1, a regulator of chromosome condensation (RCC), was significantly decreased, whereas prohibitin (PHB) (Pavir.Aa01476.1) was significantly increased (Table S1-4). UDP-glucose 4-epimerase (UGE) (Pavir.J14539.1), with a proven function in cell wall carbohydrate metabolism [45], was upregulated more than six-fold. A cell-wall-modifying xyloglucan endotransglycosylase/hydrolase (XET) (Pavir.Fa01211.1) [46] was increased 3.39-fold. These two cell-wall-related proteins were not identified in PMT (Table S1-4).

### 2.6. Phyto-Hormone Metabolism and Signaling Transduction Pathways

Four proteins involved in the metabolism of auxin and ethylene were all induced by drought stress (Pavir.Ga00273.1, Pavir.J01120.1, Pavir.J01160.1, and Pavir.Ia03739.1). Of the significantly changed proteins in the ABA-metabolic pathway, the upregulated proteins were classified as GRAM domain-containing proteins (Pavir.Ca02189.1 and Pavir.Cb00761.1), ABA-responsive elements-binding factor (ABF) (Pavir.J00256.1), and 9-cis-epoxycarotenoid dioxygenases (NCED) (Pavir.Ba03791.1) (Table S1-4).

Six calcium-binding proteins that interact with the second messenger “Ca^2+^” to transduce stress signals into plant cells were identified, five of them markedly upregulated (Pavir.Ea00612.1, Pavir.Ca00053.1, Pavir.Eb03832.1, Pavir.Ib02894.1, and Pavir.J09383.1). These proteins were identified in both CLE and PMT, or in CLE but not in PMT. The one reduced (Pavir.Da01126.1) protein was identified in PMT but not in CLE (Table S1-4).

### 2.7. Stress-Responsive Proteins

The drought treatments induced 31 abiotic/biotic stress responsive proteins. These stress proteins include six biotic stress responsive proteins (Pavir.Bb00478.1, Pavir.Fb02059.1, Pavir.Ga02124.1, Pavir.Ha00419.1, Pavir.J09667.1, and Pavir.J00406.1), five dehydrins (DHNs) (Pavir.Bb03589.1, Pavir.Ca01575.1, Pavir.Aa00887.1, Pavir.J04551.1, and Pavir.J13075.1), 13 heat shock proteins (HSP) (Pavir.Ea00289.1, Pavir.J35929.1, Pavir.J33423.1, Pavir.J24160.1, Pavir.Ia03665.1, Pavir.J40704.1, Pavir.Ib01136.1, Pavir.Ab00778.1, Pavir.J19824.1, Pavir.Fa01476.1, Pavir.Aa00282.1, Pavir.Hb01472.1, and Pavir.J21349.1), one cold stress-related protein (Pavir.J31919.1), and six other stress responsive proteins. Of them, the HSP20-like protein (Pavir.J21349.1) increased more than 9.73-fold (Table S1-4).

### 2.8. Carbohydrate Metabolism

The relative abundance level of proteins in carbohydrate metabolic pathways, such as gluconeogenesis, starch metabolism, and the biosynthesis of raffinose family oligosaccharides (RFO), were altered in response to the drought treatments. The induced proteins include malate synthase (Pavir.Gb01372.1), β amylase protein (Pavir.J18576.1), and two galactinol synthase proteins (Pavir.J07018.1 and Pavir.J40731.1), but a starch synthase (Pavir.J06822.1) was repressed under the drought-treated conditions (Table S1-4).

### 2.9. Nitric Acid Metabolism

Under moderate drought stress, three proteins involved in the biosynthesis of free amino acids were markedly upregulated, Δ1-pyrroline-5-carboxylate synthetase protein (P5CS) (Pavir.J02344.1), methionine-γ-lyase protein (MGL) (Pavir.Ib03758.1), and l-asparagine amidohydrolase (Pavir.Gb00328.1). An enzyme-catalyzing β-oxidation of fatty acids, 3-ketoacyl-CoA thiolase-2 (KAT2/PED1/PKT3) (Pavir.J16366.1) which is involved in ABA signaling, was also significant increased (Table S1-4).

## 3. Discussion

Among all the drought tolerance mechanisms, an increased ABA content in leaves has been shown to play a key role in activating signaling pathways that control stomatal closure, thus reducing transpirational water loss [47,48].

During the 20 days of drought treatment period, the switchgrass leaves showed a gradual decline in stomatal conductance and transpiration rates, which are indications of a reduced stomatal aperture. This prediction of stomatal behavior is supported by the upregulation of PHB (Pavir.Aa01476.1), which regulates the level of nitric oxide accumulation that induces stomatal closure and thus enhances the adaptive plant responses against drought stress [49,50].

**Figure 2:**
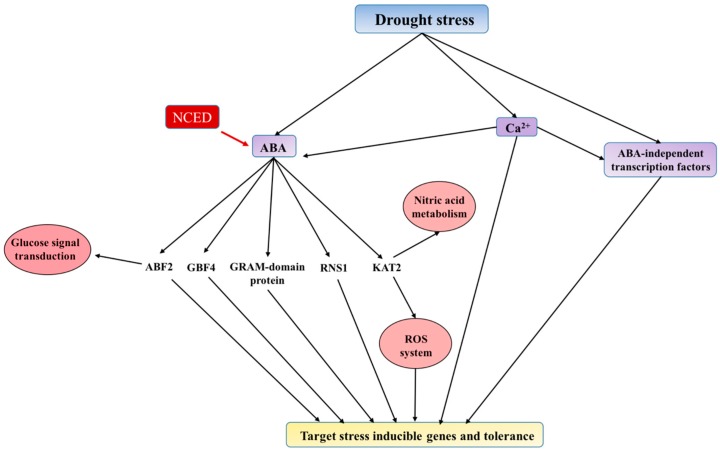
Schematic of the drought-induced signaling pathway based on proteome changes in switchgrass leaves. The biosynthesis of abscisic acid (ABA) was increased due to the elevated level of 9-*cis*-epoxycarotenoid dioxygenases (NCED) protein in drought-treated leaves. The elevated ABA level concurs with the induction of several ABA-responsive transcription factors, such as ABF2 (ABA-responsive elements-binding factor 2), GBF4 (G-box binding factor 4), GRAM, and ABA-responsive proteins including RNS (secreted ribonuclease) and KAT2 (3-ketoacyl-CoA thiolase-2). The ABA-independent signal transduction pathway appears to also play a role in drought-induced molecular regulation in switchgrass leaves. Several signal transduction processes may involve a second messenger (Ca^2+^).

Changes in protein expression support the elevated biosynthesis of ABA and the induction of ABA-mediated signal transduction pathways during the drought treatment period (Figure 2). In the ABA biosynthesis pathway, 9-*cis*-epoxycarotenoid dioxygenase (NCED) catalyzes the step to convert 9-*cis*-xanthophylls to xanthoxin, which is the direct precursor of ABA [51]. The regulatory role of NCED in ABA biosynthesis in leaves under stress conditions has been clearly demonstrated in many studies, showing that the abundance of NCED proteins is directly correlated with ABA content [51,52,53,54,55]. The same metabolic changes may have occurred in switchgrass leaves where the significant increase (4.5-fold) of an NCED protein (Pavir.Ba03791.1) may result in an elevated ABA content in the drought-treated leaves (Table S1-4).

In the ABA-dependent signaling pathway, bZIP transcription factors is one of the major families that have been described to be associated with plant responses to stress conditions [44]. In this study, two members of bZIP proteins, GBF (G-box binding factor) (Pavir.Ea03718.1) and ABF (ABA-responsive elements-binding factor) (Pavir.J00256.1), were significantly increased under drought stress. The overexpression of ABF can alter ABA sensitivity, dehydration tolerance, and the expression levels of ABA/stress-regulated genes [56]. Furthermore, the GBF and ABF protein, and an ABA-responsive GRAM domain-containing protein (Pavir.Cb00761.1) were identified as drought-induced proteins in both CLE and PMT samples, which validates the high confidence of these significant proteins. Taken together, we have shown for the first time that the ABA-dependent pathway are regulated at protein level, which in turn may have a significant role in activating the transcription of drought tolerance genes in switchgrass.

Ribonuclease S1 (RNS1) (Pavir.Fa00890.1) plays a very important part in both wound- and ABA-responsive signaling pathways, and RNS1 itself is a target for post-transcriptional regulation by ABA [57]. The upregulated enzyme 3-ketoacyl-CoA thiolase-2 (KAT2/PED1/PKT3) (Pavir.J16366.1) has an important role in regulating reactive oxygen species (ROS) production in response to ABA [58]. In addition, three proteins annotated to the regulatory components of ABA receptor 3 (Pavir.Ab01039.1, Pavir.Ca00496.1, and Pavir.Cb01723.1) showed varied changes (0.78–1.48-fold), but none of them passed the threshold criteria for significantly changed proteins in this study. These results indicate a very dynamic adjustment system regulating the expression of proteins in ABA biosynthesis and signaling pathways, which in turn modulates the activation of drought tolerance mechanism in switchgrass leaves (Figure 2).

Environmental stimuli usually require a second messenger, such as Ca^2+^, to transduce the signals into a plant cell. Under stress conditions, calcium-binding proteins (e.g., calmodulin or calmodulin-related protein) are induced in response to elevated levels of free Ca^2+^ in cells, and then they, in turn, activate signal transduction pathways with an impact on the activity of a variety of target enzymes [59,60,61,62,63,64,65,66,67,68]. The dynamic changes in the isoforms of these calcium-binding proteins quantified in this study represent the complex network of drought stress-induced signal transduction in switchgrass (Figure 2).

The drought-induced metabolic rearrangement is one of the major components for plants to acquire tolerance to stress conditions. Soluble sugars can accumulate to function as osmolytes to maintain cell turgor and have the ability to protect membranes and proteins from stress damage [69,70,71]. In the drought-treated switchgrass leaves, the induced proteins include malate synthase (Pavir.Gb01372.1), which is a key enzyme in the glyoxylate cycle for the regeneration of glucose from organic acids (Table S1-4). Maruyama et al. detected an increased level of malate synthase transcripts in rice plants subjected to drought stress, and their data implied that regulation of the glyoxylate cycle may be involved in glucose accumulation in response to dehydration in rice [69]. In the starch metabolic pathway, two proteins showed a significant alteration under drought treatment condition: the downregulated starch synthase protein (Pavir.J06822.1), which participates in starch biosynthesis, and the upregulated β amylase protein (Pavir.J18576.1) involved in the hydrolysis of starch into sugars (Table S1-4). Starch is the main form of carbohydrate storage in most plants and can be rapidly mobilized into soluble sugars. Drought and salt stress generally lead to an active conversion of starch into soluble sugars in leaves [71,72,73].

Plants experiencing environmental stress like cold, heat, drought, or salinity accumulate raffinose family oligosaccharides (RFO) in leaves [71,73,74,75,76,77,78,79]. These sugars have been implicated in membrane protection and radical scavenging [80,81]. In this study, two galactinol synthase proteins (Pavir.J07018.1 and Pavir.J40731.1) were induced in drought-treated leaves (Table S1-4), and these enzymes catalyze formation of galactinol from myo-inositol and UDP-galactose in the biosynthesis of RFO [82]. In summary, the drought-induced proteome changes seem to favor accumulation of soluble sugars, which might serve a role in protecting against cellular dehydration under drought treatment conditions.

Additionally, proteins associated with the biosynthesis of free amino acids were markedly upregulated in drought-treated leaves, which include ∆1-pyrroline-5-carboxylate synthetase protein (P5CS) (Pavir.J02344.1), the rate-limiting enzyme in proline biosynthesis, and methionine-γ-lyase protein (MGL) (Pavir.Ib03758.1), which is a precursor in isoleucine (Ile) biosynthesis (Table S1-4). Accumulation of proline (Pro) and branched-chain amino acids is commonly observed in plants subjected to osmotic stress [83,84]. Proline can serve as a free radical scavenger to overcome the oxidative stress by abiotic stress, and the accumulation of this amino acid enhances the ability of plants to grow in water-restricted or saline environments [85]. The accumulation of free isoleucine was induced in response to drought stress in *A. thaliana* [86]. The activation of these biosynthesis pathways leading to proline and isoleucine accumulation may also serve a critical role in amino acid homeostasis in drought-treated switchgrass leaves.

In the drought-treated leaves, the downregulated expression of a regulator of chromosome condensation (RCC; Pavir.Gb00127.1) may have affected the cell division, since it can bind to chromatin and generate a Ras-related nuclear protein (RAN)-guanosine triphosphate (GTP)/RAN-guanosine diphosphate (GDP) (Ran-GTP/Ran-GDP) gradient across the nuclear envelope that is required both to drive nucleocytoplasmic transport and to regulate processes associated with progression of the cell cycle and mitosis [87,88]. This might be a mechanism underlying the smaller leaf areas on drought-treated plants. Additionally, the elevated level of xyloglucan endotransglycosylase (XET) (Pavir.Fa01211.1) may assist in the process of cell wall remodeling with an impact on strengthening the wall layers and protecting mesophyll cells against physiological dehydration stress [89].

## 4. Materials and Methods

As described in Figure 1, the experiment is comprised of four major steps: drought treatments, protein sample preparation, proteomics analysis, and functional pathway classification of the drought-induced leaf proteomes.

### 4.1. Construction of a “Sandwich” Drought Treatment System

The “sandwich” treatment system was structured to simulate the process of a gradual decline in water content in the surface soil during drought under field conditions. It is comprised of double PVC pipes (an outer pipe and an inner pipe), a PVC sewer and drain coupling, and a PVC sewer and drain cap (Steinhouse Supply Company, Nashville, TN, USA). A fiberglass screen (New York Wire^®^, Grand Island, NY, USA) was placed inside the inner pipe, which assisted when pulling out the plants for checking root length.

The “sandwich” treatment system is divided into three layers: garden soil (25 m), perlite (15 cm), and garden soil (30 cm). The garden soil and perlite were products of Scotts Miracle-Gro Company (Marysville, OH, USA). After withholding water, the top layer of soil becomes drier gradually as the middle perlite layer drains the water quickly and cuts off moisture movement upward, and the moist bottom soil layer serves to induce root growth downward. The water depletion process in the top layer would induce gradual drought stress on plants. A 200SS WATERMARK Soil Moisture Sensor (IRROMETER Company Inc., Riverside, CA, USA) was placed at the bottom of the top soil layer in each growth tube.

### 4.2. Preparation of Seedling Plants

Switchgrass “Alamo” seeds were surface disinfected in 50% household bleach followed by three rinses in deionized water. Seeds were germinated in Magenta boxes partially filled with water and placed on an incubator shaker (50 rpm) at 25 °C for three days. Germinating seeds bearing 1 cm long radicals were transferred into seed cubes (Smithers-Oasis Company, Kent, OH, USA). These seedlings were watered every three days until they had grown to the three-leaf stage. At that stage, they were transplanted into the “sandwich” system and maintained in an open-roofed greenhouse at ambient temperature. The moisture content of the growing medium was maintained (soil water tension <0.01 MPa) until seedling roots reached the perlite layer. Each biological replicate contained 10 tubes each growing two plants, and four biological replicates were set up for drought-treated and non-treated control groups. A randomized block design was used in this study.

### 4.3. Drought Treatment and Physiological Measurements

Two weeks after transplanting, the root length was evaluated every three days. Three samples from each replicate group were selected, randomly, in each inspection. Once the longest roots reached the perlite layer, drought treatment was initiated by withholding water to these test plants. The control groups received normal watering at the rate of 4 L of water every three days for each “sandwich” system. The drought treatment was initiated on 25 April and ended on 15 May 2013.

Leaf photosynthetic rate, stomatal conductance, and transpiration rate were measured using a LI-COR 6400 Portable Photosynthesis System (Li-Cor Inc., Lincoln, NE, USA). Two fully expanded young leaves randomly selected from each plant were measured between 10:00 am and 3:00 pm. Light in the leaf chamber was set at 2000 µmol photons/m^2^/s. Water use efficiency (WUE) was calculated by WUE = leaf photosynthetic rate (Pn)/transpiration (Tr) [43]. Soil water tension was recorded daily. Plant height was collected before ($H_{b}$) and after ($H_{a}$) the drought treatment and was measured from the bottom of the tiller (start point) to the bottom of the latest node (end point). Relative plant height $(H_{r}=H_{b}-H_{a})\;$was used to compare the difference of plant relative growth rate during the drought treatment. Fresh weight ($W_{f}$) of leaves were measured at harvest, and they were dried at 70 °C for three days until a constant dry weight ($W_{d}$). Relative water content was calculated by $W_{r}=(W_{f}-W_{d})/W_{f}$. Data analysis was performed using PROC GLM procedure of SAS software (Version: 9.3. SAS Inc., Cary, NC, USA). The effect of drought treatments was analyzed using a randomized block design analysis of variance (ANOVA). When a significant effect of drought treatment was detected, least significant difference (LSD) was used for multiple comparisons.

### 4.4. Tissue Harvest and Preparation of Protein Samples

Twenty days after water withholding, when the top-layer soil moisture declined to below 0.05 MPa and stomatal conductance, respiration, and water use efficiency of leaves showed a significant difference between drought-treated and non-treated control groups, plants were considered to have activated the drought-induced physiological process. The top three fully expanded leaves were cut into approximately 2 cm long pieces, wrapped with aluminum foil, frozen in liquid nitrogen, and stored at −80 °C until protein extraction.

Frozen samples were ground into a fine powder under liquid nitrogen using a Retsch Mixer Mill MM 400 (Retsch GmbH, Haan, Germany). Protein extraction followed a previously described protocol [18]. Briefly, leaf tissue powder was washed sequentially in 10% trichloroacetic acid (TCA) in acetone, 80% methanol in 0.1 M ammonium acetate, and 80% acetone with centrifugation to pellet the powder after each step. Protein was then extracted in a phenol (pH 8.0) and dense sodium dodecyl sulfate (SDS) buffer (30% sucrose, 2% SDS, 5% β-mercaptoethanol (*v*/*w*) in 0.1 M Tris-HCl, pH 8.0). After incubation at 4 °C for 2 h, the mixture was centrifuged at 16,000× *g* at 4 °C for 20 min. Protein in the upper phenol phase was precipitated in 0.1 M ammonium acetate in methanol after incubation overnight at −20 °C. After washes in methanol and then acetone, the air-dried protein pellets were wetted in a buffer containing 500 mM triethylammonium bicarbonate (TEAB), 2 M urea, 0.1% sodium dodecyl sulfate (SDS), and a protease inhibitor cocktail for plant cell and tissue extracts (100× dilution in the extraction buffer) (Part #9599; Sigma, St. Louis, MO, USA).

For enrichment of low-abundance proteins, the individual protein extracts were processed using a ProteoMiner Protein Enrichment kit (Bio-Rad, Hercules, CA, USA). One milliliter of each protein sample was added to the ProteoMiner columns. Proteins were bound to beads after shaking in the columns using a Mini LabRoller overnight at room temperature. Columns were then washed three times with a wash buffer (150 mM NaCl, 10 mM NaH_2_PO_4_, pH 7.4). Then, the columns were incubated at room temperature for 15 min in rehydrated elution reagent (8 M urea, 2% 3-((3-cholamidopropyl) dimethylammonium)-1-propanesulfonate (CHAPS) and 5% acetic acid) before eluting the proteins. Proteins were concentrated using 5 KDa Corning Spin-X UF centrifugal concentrator (Sigma, St. Louis, MO, USA).

Protein concentration was determined using a Bradford Assay Kit (Bio-Rad). Protein quality was examined by separating 15 μg of proteins on 10%–20% precast Criterio TGX polyacrylamide gels (Bio-Rad).

### 4.5. Isobaric Tags for Relative and Absolute Quantification (iTRAQ) Labeling and Mass Spectrometry Analysis

For iTRAQ labeling, protein samples containing 100 μg protein each were diluted using a buffer containing 500 mM TEAB, 0.1% SDS, and the same protease inhibitor as described above at the same concentration to reduce urea concentration to below 1 M. Then the protein sample was processed following the instructions of the 8-plex iTRAQ labeling kit [21]. Protein tryptic digestion was conducted using sequence grade modified trypsin (Promega, Madison, WI, USA) after incubation at 37 °C for 16 h. The control samples were labeled with tags 113, 115, 117, and 118 and the treated samples with 114, 116, 119, and 121. After combining all the labeled samples, unbound tags and SDS were removed through cation exchange cartridge (AB SCIEX). Salts and other impurities were removed using reverse-phase (RP) solid-phase extraction procedure involving 1-cm^3^, 50-mg Sep-Pak C18 cartridges following the manufacturer’s instructions (Waters; Milford, MA, USA). Peptides were eluted in 500 μL 50% (*v*/*v*) acetonitrile with 0.1% trifluoroacetic acid (TFA). Samples were dried at reduced pressure using a CentiVac Concentrator (labConco, Kansas City, MO, USA).

The peptide samples were subjected to a first dimension of high-pH Ultra Performance Liquid. Chromatography (UPLC) separation using an Acquity UPLC System (Waters) coupled with a robotic fraction collector (Probot; Dionex, Sunnyvale, CA, USA) [21]. One hundred micrograms of the multiplexed sample were injected and fractionated into 48 fractions in a 96-well plate. The 48 fractions were concatenated to yield 22 samples as follows: samples 1–4 and 45–48 were combined to yield two 2nd dimension fractions (samples 1–4 not analyzed in 2nd dimension); then for the remaining samples (5–44), every 20th fraction was combined. For the low-pH second dimension, low-pH RP chromatography was employed. Dried samples were reconstituted with 15 μL of 2% acetonitrile with 0.5% formic acid. Nano-LC separations of tryptic peptides were performed as described previously. The eluent from the analytical column was delivered to the LTQ-Orbitrap Elite (Thermo-Fisher Scientific, Waltham, MA, USA) via a “Plug and Play” nano ion source (CorSolutions LLC, Ithaca, NY, USA). The mass spectrometer was externally calibrated across the *m*/*z* range from 375–1800 with Ultramark 1621 for the Fourier transform (FT) mass analyzer, and individual runs were internally calibrated with the background polysiloxane ion at *m*/*z* 445.1200025 as a lock mass [24,90,91].

The Orbitrap Elite was operated in the positive ion mode with nanosource voltage set at 1.7 kV and capillary temperature at 250 °C. A parallel data-dependent acquisition (DDA) mode was used to obtain one MS survey scan with the FT mass analyzer, followed by isolation and fragmentation of the 15 most abundant, multiply-charged precursor ions with a threshold ion count higher than 50,000 in both the LTQ mass analyzer and the high energy collisionally induced dissociation (HCD)-based FT mass analyzer at a resolution of 15,000 full width at half maximum (FWHM) and *m*/*z* 400. MS survey scans were acquired with resolution set at 60,000 across the survey scan range (*m*/*z* 375–1800). Dynamic exclusion was utilized with repeat count set to 1 with a 40 s repeat duration; exclusion list size was set to 500, 20 s exclusion duration, and low and high exclusion mass widths set to 1.5. Fragmentation parameters were set with isolation width at 1.5 *m*/*z*, normalized collision energy at 37%, and activation Q at 0.25. Activation time for HCD analysis was 0.1 min. All data were acquired using XCalibur 2.1 (Thermo-Fisher Scientific) [18,24]. Proteins were identified using the MS data to query the switchgrass annotated database (http://www.phytozome.net/) via Mascot v2.3.02 (Matrix Sciences, Boston, MA, USA). The mass spectrometry proteomics data have been deposited to the ProteomeXchange Consortium via the PRIDE [92] partner repository with the dataset identifier PXD004675 and 10.6019/PXD004675.

### 4.6. Protein Identification and Quantification, and Statistics Analysis

For a protein to be included in the quantitative analysis, it was required that at least two unique peptides have to be identified in all eight biological samples. The intensities of reporter ions of constituent peptides were log2-transformed. Then, log2 fold values from all constituent peptides were subjected to *t*-test (general linear model procedure) followed by false discovery rate (FDR) corrections to test the statistical significance of the difference in normalized abundance of each protein between the drought-treated and control sample groups [21]. The log2 transformed abundance ratios were then fit to a normal distribution (*p* < 0.01) [93]. Two standard deviations (i.e., a 95% confidence level) of the log2 fold transformed protein abundance ratio (treated/control) were used as the cutoff for significantly changed proteins. The antilog conversion was used to represent the fold change of proteins. Statistical analyses were performed using SAS (version 9.3; SAS Institute, Cary, NC, USA) [18].

### 4.7. Functional Pathway Analysis of Drought-Induced Proteins

In the annotated switchgrass database (*Panicum virgatum v1.1*, Phytozome v11.0), each accession is associated with a unigene accession in *Arabidopsis thaliana*. The switchgrass annotated genome is not included in the database of the MapMan pathway tools. Therefore, in this study, the *A. thaliana* database in MapMan (MapMan, version 3.5.1R2, Max Planck Institute of Molecular Plant Physiology, Potsdam-Golm, Germanry) was used to develop the functional pathways [94]. Additional literature and database searches were conducted to develop the association between drought-induced proteins and drought tolerance, and highlight new discoveries using proteomics analysis.

### 4.8. Statistical Analysis

All independent experiments were repeated four times. Experimental data were presented as means and standard deviations (SD). The SAS version 9.0 software (SAS Inc., Cary, NC, USA) was used to perform the analysis of variance (ANOVA) and least significant difference (LSD) tests for the physiological data, and *t*-tests and FDR tests in the analysis of quantitative proteomics data.

## 5. Conclusions

This study has identified drought-induced changes in leaf proteomes that occurred when plants have shown significant physiological changes from drought-treated to non-treated control conditions. The identified proteins are involved in both ABA-dependent and ABA-independent signaling pathways, and diverting metabolic pathways toward increasing cellular concentrations of soluble sugars and stress-related amino acids (proline and isoleucine). The accumulation of a diverse species of stress proteins can be considered as the hallmark for switchgrass plants to acquire drought tolerance. Information provided in this paper advanced our understanding of molecular mechanisms underlying drought tolerance in C_4_ plants.

## Figures and Tables

**Table 1:** Effects of drought treatments on physiological properties of switchgrass.

Treatment		Control	Drought
Soil Water Tension (MPa)		0.00 ± 0.00 ^A,†^	0.08 ± 0.02 ^B,†^
Leaf Relative Water Content		77.35 ± 0.01 ^A^	71.08 ± 0.02 ^B^
Plant Height (cm)	0 Day drought treatment	18.31 ± 6.18 ^A^	19.08 ± 4.97 ^A^
	20 days drought treatment	43.26 ± 9.11 ^A^	39.75 ± 8.49 ^B^
	Relative plant height	24.96 ± 6.21 ^A^	20.67 ± 6.22 ^B^
Photosynthesis	Leaf photosynthetic rate (μmol CO_2_/m^2^/s)	22.96 ± 3.22 ^A^	21.69 ± 7.17 ^A^
	Stomatal conductance (mol H_2_O/m^2^/s)	0.138 ± 0.03 ^A^	0.125 ± 0.05 ^B^
	Transpiration rate (mmol H_2_O/m^−2^/s)	6.88 ± 1.11 ^A^	6.09 ± 2.15 ^B^
	Water use efficiency (μmol CO_2_/mmol H_2_O)	3.35 ± 0.20 ^A^	3.59 ± 0.25 ^B^

Data for all the measurements except plant height were collected after 20 days of the water withholding treatments. Data are presented as means ± standard deviations (SD) of four independent replicates. Within columns, means followed by the same letter are not significantly different (*p* < 0.01). Leaf relative water content (*W*_r_) was calculated using the following equation: $W_{r}=(W_{f}-W_{d})/W_{f}$, where fresh weight ($W_{f}$) was taken immediately after harvest, and dry weight was measured after drying tissues at 70 °C for three days until a constant dry weight ($W_{d}$). Plant height was measured from the bottom of the tiller (start point) to the top of the latest node (end point). ^†^ Means within columns followed by the same letter are not different at the 1% level.

**Table 2:** The number of proteins identified in the proteomes identified using the crude leaf protein extracts and ProteoMiner-enriched samples.

Protein Classification		CLE ^a^	PMT ^b^	The Number of Proteins from CLE and PMT
Proteins identified with one or more peptides	The total number of proteins	5493	4839	7006
	The number of proteins overlapped in CLE and PMT	3326		
	The number of proteins identified in CLE	2167	-	
	The number of protein identified in PMT	-	1513	
Quantified proteins with two or more peptides	The total number of proteins	4746	4134	5680
	The number of proteins overlapped in CLE and PMT	3200		
	The number of protein in CLE	1546	-	
	The number of proteins in PMT	-	934	
Differentially expressed proteins (FDR < 0.01, fold change < 0.06 or > 1.7)	The total number of proteins	205	107	257
	The number of proteins in CLE and PMT	55		
	The number of proteins in CLE	150	-	
	The number of proteins in PMT	-	52	

^a^ The number of proteins identified in the crude leaf protein extracts; ^b^ The number of proteins identified in the ProteoMiner enriched samples; CLE: Crude Leaf Extracts; PMT: ProteoMiner-treated; FDR: false discovery rate.

**Table 3:** The number of proteins identified in the crude leaf protein extracts and ProteoMiner-treated samples.

Classification		CLE ^a^	PMT ^b^	CLE and PMT ^c^
Molecular Function	Abiotic/biotic stress	72	25	116
	Cell division/cell cycle	11	7	42
	Cell organization	26	11	47
	Cell vesicle transport	21	6	31
	Development	41	16	46
	DNA repair	4	2	7
	DNA synthesis	20	15	28
	Functional enzyme	62	64	180
	Metal binding	4	1	11
	Phyto-hormone metabolism	21	11	36
	Protein and amino acids activation	15	13	35
	Protein degradation	88	39	172
	Protein post-translation	27	12	41
	Protein synthesis	61	54	209
	Protein targeting	21	22	81
	Redox balance	31	18	98
	RNA transcription/processing	113	74	212
	Signaling regulation	82	39	98
	Transport	24	35	65
Cellular Metabolism	Amino acid metabolism	39	38	91
	C1-metabolism	4	5	16
	Cell wall synthesis/modification	13	13	20
	Fermentation	3	3	6
	Glycolysis	9	12	41
	Glyoxylate cycle	1	0	10
	Lipid metabolism	22	30	51
	Major CHO metabolism	11	10	35
	Minor CHO metabolism	7	0	26
	Mitochondrial electron transport/ATP synthesis	9	9	57
	N-metabolism	2	2	7
	Nucleotide metabolism	24	14	53
	Oxidative pentose phosphate (OPP) pathway	7	3	12
	Photosystem. Calvin cycle	4	6	36
	Photosystem. Light reaction	15	10	82
	Photorespiration	3	1	14
	S-assimilation	2	2	5
	Secondary metabolism	18	29	67
	TCA cycle	8	10	52
	Tetrapyrrole synthesis	13	9	20
Others and not assigned proteins		588	264	944
Total		1546	934	3200

^a^ The number of proteins identified in the crude leaf protein extracts (CLE); ^b^ The number of proteins identified in the ProteoMiner-treated samples (PMT); ^c^ The number of proteins combining the proteomes identified in CLE and PMT.

## Supplementary Materials

Supplementary materials can be found at http://www.mdpi.com/1422-0067/17/8/1251/s1.

## References

[1] Wright L.I., Cushman J.H., Ehrenshaft A.R., McLaughlin S.B., McNabb W.A., Martin S.A., Ranney J.W., Tuskan A.G., Turhollow A.F. (1993). Biofuels Feedstock Development Program Annual Progress Report for 1992.

[2] Parrisha D.J., Fike J.H. (2005). The biology and agronomy of switchgrass for biofuels. CRC. Crit. Rev. Plant Sci..

[3] Wright L.L. (2007). Historical Perspective on How and Why Switchgrass Was Selected as a “Model” High-Potential Energy Crop.

[4] Nezhadahmadi A., Prodhan Z.H., Faruq G. (2013). Drought tolerance in wheat. Sci. World J..

[5] National Weather Service Centers for Environmental Prediction. http://www.cpc.ncep.noaa.gov/products/monitoring_and_data/topsoil.shtml.

[6] Keyser P., Harper C., Bates G., Waller J., Doxon E. (2011). Native warm-season grasses for mid-south forage production. UT Ext..

[7] Sanderson M.A., Reed R.L. (2000). Switchgrass growth and development: Water, nitrogen, and plant density effects. J. Range Manag..

[8] Vogel K.P. Improved plant & production practices for grasslands & biomass crops in the mid-continental USA. Proceedings of the DOE/USDA Biomass Feedstock Gate Review Meeting.

[9] Farooq M., Wahid A., Kobayashi N., Fujita D., Basra S.M.A. (2009). Plant drought stress: Effects, mechanisms and management. Agron. Sustain. Dev..

[10] Nonami H. (1998). Plant water relations and control of cell elongation at low water potentials. J. Plant Res..

[11] Kaya M.D., Okçub G., Ataka M., Çıkılıc Y., Kolsarıcıa Ö. (2006). Seed treatments to overcome salt and drought stress during germination in sunflower (*Helianthus annuus* L.). Eur. J. Agron..

[12] Hussain M., Malik M.A., Farooq M., Ashraf M.Y., Cheema M.A. (2008). Improving drought tolerance by exogenous application of glycinebetaine and salicylic acid in sunflower. J. Agron. Crop Sci..

[13] Liu Y., Zhang X., Tran H., Shan L., Kim J., Childs K., Ervin E.H., Frazier T., Zhao B. (2015). Assessment of drought tolerance of 49 switchgrass (*Panicum virgatum*) genotypes using physiological and morphological parameters. Biotechnol. Biofuels.

[14] Alberts B., Johnson A., Lewis J. (2002). Molecular Biology of the Cell.

[15] Barkla B.J., Castellanos-Cervantes T., de León J.L.D., Matros A., Mock H.P., Perez-Alfocea F., Salekdeh G.H., Witzel K., Zörb C. (2013). Elucidation of salt stress defense and tolerance mechanisms of crop plants using proteomics—current achievements and perspectives. Proteomics.

[16] Ghosh D., Xu J. (2014). Abiotic stress responses in plant roots: A proteomics perspective. Front. Plant Sci..

[17] Ngara R., Ndimba B.K. (2014). Understanding the complex nature of salinity and drought-stress response in cereals using proteomics technologies. Proteomics.

[18] Okekeogbu I., Ye Z., Sangireddy S., Li H., Bhatti S., Hui D., Zhou S., Howe K., Fish T., Yang Y. (2014). Effect of aluminum treatment on proteomes of radicles of seeds derived from AL-treated tomato plants. Proteomes.

[19] Zhou S., Sauve R., Fish T., Thannhauser T.W. (2009). Salt-induced and Salt-suppressed Proteins in Tomato Leaves. J. Am. Soc. Hortic. Sci..

[20] Zhou S., Sauvé R., Liu Z., Reddy S., Bhatti S. (2011). Heat-induced proteome changes in tomato leaves. J. Am. Soc. Hortic. Sci..

[21] Zhou S., Palmer M., Zhou J., Bhatti S., Howe K., Fish T., Thannhauser T.W. (2013). Differential root proteome expression in tomato genotypes with contrasting drought tolerance exposed to dehydration. J. Am. Soc. Hort. Sci..

[22] Pottiez G., Wiederin J., Fox H.S., Ciborowski P. (2012). Comparison of 4-plex to 8-plex iTRAQ quantitative measurements of proteins in human plasma samples. J. Proteome Res..

[23] Chen X., Walker A.K., Strahler J.R., Simon E.S., Tomanicek-Volk S.L., Nelson B.B., Hurley M.C., Ernst S., Williams J., Andrews P.C. (2006). Organellar proteomics: Analysis of pancreatic zymogen granule membranes. Mol. Cell. Proteom..

[24] Yang Q.S., Wu J.H., Li C.Y., Wei Y.R., Sheng O., Hu C.H., Kuang R.-B., Huang Y.-H., Peng X.-X., McCardle J. (2012). Quantitative proteomic analysis reveals that antioxidation mechanisms contribute to cold tolerance in plantain (*Musa paradisiaca* L.; ABB Group) seedlings. Mol. Cell. Proteom..

[25] Lan P., Li W.F., Wen T.N., Shiau J.Y., Wu Y.C., Lin W.D., Schmidt W. (2011). iTRAQ protein profile analysis of arabidopsis roots reveals new aspects critical for iron homeostasis. Plant Physiol..

[26] Redding A.M., Mukhopadhyay A., Joyner D.C., Hazen T.C., Keasling J.D. (2006). Study of nitrate stress in desulfovibrio vulgaris hildenborough using iTRAQ proteomics. Brief. Funct. Genom. Proteom..

[27] Nveawiah-Yoho P., Zhou J., Palmer M., Sauve R., Zhou S., Howe K.J., Fish T., Thannhauser T.W. (2013). Identification of proteins for salt tolerance using a comparative proteomics analysis of tomato accessions with contrasting salt tolerance. J. Am. Soc. Hort. Sci..

[28] Nveawiah-Yoho P. (2012). Mechanisms for Salt Tolerance and Susceptibility in Tomato. Ph.D. Thesis.

[29] Ali G.M., Komatsu S. (2006). Proteomic analysis of rice leaf sheath during drought stress. J. Proteom. Res..

[30] Ke Y., Han G., He H., Li J. (2009). Differential regulation of proteins and phosphoproteins in rice under drought stress. Biochem. Biophys. Res. Commun..

[31] Salekdeh G., Siopongco J., Wade L., Ghareyazie B., Bennett J. (2002). Proteomic analysis of rice leaves during drought stress and recovery. Proteomics.

[32] Salekdeh G., Siopongco J., Wade L., Ghareyazie B., Bennett J. (2002). A proteomic approach to analyzing drought- and salt-responsiveness in rice. F. Crop. Res..

[33] Shu L., Ding W., Wu J., Feng F., Luo L. (2010). Proteomic analysis of rice leaves shows the different regulations to osmotic stress and stress signals. J. Integr. Plant Biol..

[34] Xiong J., Fu B., Xu H., Li Y. (2010). Proteomic analysis of PEG-simulated drought stress-responsive proteins of rice leaves using a pyramiding rice line at the seedling stage. Bot. Stud..

[35] De Vienne D., Leonardi A., Damerval C., Zivy M. (1999). Genetics of proteome variation for QTL characterization: Application to drought-stress responses in maize. J. Exp. Bot..

[36] Mohammadkhani N., Heidari R. (2008). Effects of drought stress on soluble proteins in two maize varieties. Turkish J. Biol..

[37] Riccardi F., Gazeau P., de Vienne D., Zivy M. (1998). Protein changes in response to progressive water deficit in maize. Plant Physiol..

[38] McCabe M.S., Garratt L.C., Schepers F., Jordi W.J., Stoopen G.M., Davelaar E., van Rhijn J.H., Power J.B., Davey M.R. (2001). Effects of P(SAG12)-IPT gene expression on development and senescence in transgenic lettuce. Plant Physiol..

[39] Widjaja I., Naumann K., Roth U., Wolf N., Mackey D., Dangl J.L., Scheel D., Lee J. (2009). Combining subproteome enrichment and Rubisco depletion enables identification of low abundance proteins differentially regulated during plant defense. Proteomics.

[40] Fasoli E., D’Amato A., Kravchuk A.V., Boschetti E., Bachi A., Righetti P.G. (2011). Popeye strikes again: The deep proteome of spinach leaves. J. Proteom..

[41] Von Toerne C., Kahle M., Schäfer A., Ispiryan R., Blindert M., Hrabe De Angelis M., Neschen S., Ueffing M., Hauck S.M. (2013). Apoe, Mbl2, and Psp plasma protein levels correlate with diabetic phenotype in NZO mice-An optimized rapid workflow for SRM-based quantification. J. Proteome Res..

[42] Fonslow B.R., Carvalho P.C., Academia K., Freeby S., Xu T., Nakorchevsky A., Paulus A., Yates J.R. (2011). Improvements in proteomic metrics of low abundance proteins through proteome equalization using ProteoMiner prior to MudPIT. J. Proteome Res..

[43] Polley H.W. (2002). Implications of atmospheric and climatic change for crop yield and water use efficiency. Crop Sci..

[44] Finkelstein R.R., Lynch T.J. (2000). The *Arabidopsis* abscisic acid response gene ABI5 encodes a basic leucine zipper transcription factor. Plant Cell.

[45] Rösti J., Barton C.J., Albrecht S., Dupree P., Pauly M., Findlay K., Roberts K., Seifert G.J. (2007). UDP-glucose 4-epimerase isoforms UGE2 and UGE4 cooperate in providing UDP-galactose for cell wall biosynthesis and growth of *Arabidopsis thaliana*. Plant Cell.

[46] Uozu S., Tanaka-Ueguchi M., Kitano H., Hattori K., Matsuoka M. (2000). Characterization of XET-related genes of rice. Plant Physiol..

[47] Nambara E., Marion-Poll A. (2005). Abscisic acid biosynthesis and catabolism. Annu. Rev. Plant Biol..

[48] Leckie C.P., McAinsh M.R., Allen G.J., Sanders D., Hetherington A.M. (1998). Abscisic acid-induced stomatal closure mediated by cyclic ADP-ribose. Proc. Natl. Acad. Sci. USA.

[49] Wang Y., Ries A., Wu K., Yang A., Crawford N.M. (2010). The *Arabidopsis* prohibitin gene PHB3 functions in nitric oxide-mediated responses and in hydrogen peroxide-induced nitric oxide accumulation. Plant Cell.

[50] García-Mata C., Lamattina L. (2001). Nitric oxide induces stomatal closure and enhances the adaptive plant responses against drought stress. Plant Physiol..

[51] Lefebvre V., North H., Frey A., Sotta B., Seo M., Okamoto M., Nambara E., Marion-Poll A. (2006). Functional analysis of *Arabidopsis* NCED6 and NCED9 genes indicates that ABA synthesized in the endosperm is involved in the induction of seed dormancy. Plant J..

[52] Huo H., Dahal P., Kunusoth K., McCallum C.M., Bradford K.J. (2013). Expression of 9-*cis*-EPOXYCAROTENOID DIOXYGENASE4 is essential for thermoinhibition of lettuce seed germination but not for seed development or stress tolerance. Plant Cell.

[53] Tan B.C., Joseph L.M., Deng W.T., Liu L., Li Q.B., Cline K., McCarty D.R. (2003). Molecular characterization of the Arabidopsis 9-*cis* epoxycarotenoid dioxygenase gene family. Plant J..

[54] Seo M., Kanno Y., Frey A., North H.M., Marion-Poll A. (2016). Dissection of Arabidopsis NCED9 promoter regulatory regions reveals a role for ABA synthesized in embryos in the regulation of GA-dependent seed germination. Plant Sci..

[55] Iuchi S., Kobayashi M., Taji T., Naramoto M., Seki M., Kato T. (2001). Regulation of drought tolerance by gene manipulation of 9-*cis*-epoxycarotenoid dioxygenase, a key enzyme in abscisic acid biosynthesis in *Arabidopsis*. Plant J..

[56] Kim S., Kang J., Cho D.-I., Park J.H., Kim S.Y. (2004). ABF2, an ABRE-binding bZIP factor, is an essential component of glucose signaling and its overexpression affects multiple stress tolerance. Plant J..

[57] Hillwig M.S., LeBrasseur N.D., Green P.J., MacIntosh G.C. (2008). Impact of transcriptional, ABA-dependent, and ABA-independent pathways on wounding regulation of RNS1 expression. Mol. Genet. Genom..

[58] Jiang T., Zhang X.F., Wang X.F., Zhang D.P. (2011). Arabidopsis 3-Ketoacyl-CoA Thiolase-2 (KAT2), an enzyme of fatty acid β-Oxidation, is involved in ABA signal transduction. Plant Cell Physiol..

[59] Knight M.R., Campbell A.K., Smith S.M., Trewavas A.J. (1991). Transgenic plant aequorin reports the effects of touch and cold-shock and elicitors on cytoplasmic calcium. Nature.

[60] Knight M., Read N., Campbell A., Trewavas A. (1993). Imaging calcium dynamics in living plants using semi-synthetic recombinant aequorins. J. Cell Biol..

[61] Knight M.R., Smith S.M., Trewavas A.J. (1992). Wind-induced plant motion immediately increases cytosolic calcium. Proc. Natl. Acad. Sci. USA.

[62] Braam J. (1992). Regulated expression of the calmodulin-related TCH genes in cultured *Arabidopsis* cells: Induction by calcium and heat shock. Proc. Natl. Acad. Sci. USA.

[63] Haley A.N.N., Russell A.J., Wood N., Allan A.C., Knight M., Campbell A.K., Trewavas A.J., Ryan C.A., Lamb C.J., Jagendorf A.T. (1995). Effects of mechanical signaling on plant cell cytosolic calcium. Proc. Natl. Acad. Sci. USA.

[64] Klee C., Vanaman T. (1982). Calmodulin. Adv. Protein Chem..

[65] Roberts D., Lukas T., Watterson D. (1986). Structure, function, and mechanisms of action of calmodulin. CRC Crit. Rev. Plant Sci..

[66] Cohen P., Klee C. (1988). Calmodulin.

[67] Allan E., Hepler P., Stumpf P., Lonn E. (1989). Calmodulin and calcium-binding proteins. The Biochemistry of Plants.

[68] Roberts D.M., Harmon A.C. (1992). Calcium modulated proteins targets of intracellular calcium signals in higher plants. Annu. Rev. Plant Physiol. Plant Mol. Biol..

[69] Maruyama K., Urano K., Yoshiwara K., Morishita Y., Sakurai N., Suzuki H., Kojima M., Sakakibara H., Shibata D., Saito K. (2014). Integrated analysis of the effects of cold and dehydration on rice metabolites, phytohormones, and gene transcripts. Plant Physiol..

[70] Madden T., Bally M., Hope M., Cullis P., Schieren H., Janoff A. (1985). Protection of large unilamellar vesicles by trehalose during dehydration: Retention of vesicle contents. Biochim. Biophys. Acta.

[71] Kaplan F., Guy C.L. (2004). β-amylase induction and the protective role of maltose during temperature shock. Plant Physiol..

[72] Basu P.S., Ali M., Chaturvedi S.K. (2007). Osmotic adjustment increases water uptake, remobilization of assimilates and maintains photosynthesis in chickpea under drought. Indian J. Exp. Biol..

[73] Kempa S., Krasensky J., Dal Santo S., Kopka J., Jonak C. (2008). A Central Role of Abscisic Acid in Stress-Regulated Carbohydrate Metabolism. PLoS ONE.

[74] Castonguay Y., Nadeau P. (1998). Enzymatic control of soluble carbohydrate accumulation in cold-acclimated crowns of alfalfa. Crop Sci..

[75] Gilmour S., Sebolt A., Salazar M., Everard J., Thomashow M. (2000). Overexpression of the Arabidopsis CBF3 transcriptional activator mimics multiple biochemical changes associated with cold acclimation. Plant Physiol..

[76] Taji T., Ohsumi C., Iuchi S., Seki M., Kasuga M., Kobayashi M., Yamaguchi-Shinozaki K., Shinozaki K. (2002). Important roles of drought- and cold-inducible genes for galactinol synthase in stress tolerance in Arabidopsis thaliana. Plant J..

[77] Cook D., Fowler S., Fiehn O., Thomashow M.F. (2004). A prominent role for the CBF cold response pathway in conFigure uring the low-temperature metabolome of Arabidopsis. Proc. Natl. Acad. Sci. USA.

[78] Peters S., Mundree S., Thomson J., Farrant J., Keller F. (2007). Protection mechanisms in the resurrection plant Xerophyta viscosa (Baker): Both sucrose and raffinose family oligosaccharides (RFOs) accumulate in leaves in response to water deficit. J. Exp. Bot..

[79] Usadel B., Bläsing O.E., Gibon Y., Poree F., Höhne M., Günter M., Trethewey R., Kamlage B., Poorter H., Stitt M. (2008). Multilevel genomic analysis of the response of transcripts, enzyme activities and metabolites in *Arabidopsis* rosettes to a progressive decrease of temperature in the non-freezing range. Plant Cell Environ..

[80] Hincha D.K. (2003). Effects of calcium-induced aggregation on the physical stability of liposomes containing plant glycolipids. Biochim. Biophys. Acta.

[81] Nishizawa A., Yabuta Y., Shigeoka S. (2008). Galactinol and raffinose constitute a novel function to protect plants from oxidative damage. Plant Physiol..

[82] Peterbauer T., Richter A. (2001). Biochemistry and physiology of raffinose family oligosaccharides and galactosyl cyclitols in seeds. Seed Sci. Res..

[83] Rhodes D., Handa S., Bressan R. (1986). Metabolic changes associated with adaptation of plant cells to water stress. Plant Physiol..

[84] Girousse C., Bournoville R., Bonnemain J.L. (1996). Water deficit-induced changes in concentrations in proline and some other amino acids in the phloem sap of alfalfa. Plant Physiol..

[85] Hong Z. (2000). Removal of feedback inhibition of delta 1-pyrroline-5-carboxylate synthetase results in increased proline accumulation and protection of plants from osmotic stress. Plant Physiol..

[86] Joshi V., Jander G. (2009). *Arabidopsis* methionine garmma-lyase is regulated according to isoleucine biosynthesis needs but Plays a subordinate. Plant Physiol..

[87] Bischoff F.R., Ponstingl H. (1991). Catalysis of guanine nucleotide exchange on Ran by the mitotic regulator RCC1. Nature.

[88] Nemergut M.E., Lindsay M.E., Brownawell A.M., Macara I.G. (2002). Ran-binding protein 3 links Crm1 to the Ran guanine nucleotide exchange factor. J. Biol. Chem..

[89] Cho S.K., Kim J.E., Park J.A., Eom T.J., Kim W.T. (2006). Constitutive expression of abiotic stress-inducible hot pepper CaXTH3, which encodes a xyloglucan endotransglucosylase/hydrolase homolog, improves drought and salt tolerance in transgenic Arabidopsis plants. FEBS Lett..

[90] Thannhauser T., Shen M., Sherwood R., Howe K., Fish T., Yang Y., Chen W., Zhang S. (2013). A workflow for large-scale empirical identification of cell wall N-linked glycoproteins of tomato (*Solanum lycopersicum*). Electrophoresis.

[91] Yang Y., Qiang X., Owsiany K., Zhang S., Thannhauser T.W., Li L. (2011). Evaluation of different multidimensional LC-MS/MS pipelines for iTRAQ-based proteomic analysis of potato tubers in response to cold storage. J. Proteome Res..

[92] Vizcaíno J.A., Csordas A., del-Toro N., Dianes J.A., Griss J., Lavidas I., Mayer G., Perez-Riverol Y., Reisinger F., Ternent T. (2016). 2016 update of the PRIDE database and related tools. Nucleic Acids Res..

[93] Zhou S., Okekeogbu I., Sangireddy S., Ye Z., Li H., Bhatti S., Hui D., Mcdonald D.W., Yang Y., Giri S. (2016). Proteome modification in tomato plants upon long-term aluminum treatment. J. Proteome Res..

[94] MapMan, Version 3.5.1R2 2015. http://mapman.gabipd.org.

