# Supplementary material for: Drought-Induced Leaf Proteome Changes in Switchgrass Seedlings
Source: Int J Mol Sci. 2016 Aug 2;17(8):1251. doi: 10.3390/ijms17081251 (PMC5000649; doi:10.3390/ijms17081251)
Supplement: Supplementary file 1 [file ijms-17-01251-s001.zip › ijms-139535-supp-fig.pdf]

Supplementary Materials: Drought-Induced Leaf Proteome Changes in Switchgrass Seedlings

Zhujia Ye, Sasikiran Sangireddy, Ikenna Okekeogbu, Suping Zhou, Chih-li Yu, Dafeng Hui, Kevin J. Howe, Tara Fish and Theodore W. Thannhauser

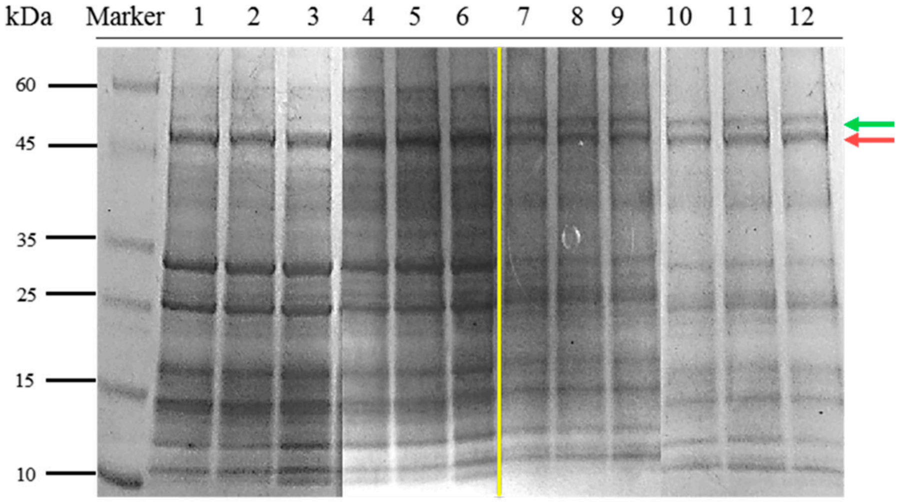

Figure S1. Images of *one-dimensional gel electrophoresis of switchgrass leaf proteins showing the effects of* ProteoMiner enrichment in the removal of highly abundant proteins. Lanes "1–6": crude protein extracts from leaf samples; lanes "7–12": protein samples treated with ProteoMiner; lanes "1–3" and "7–9": proteins from non-drought treated control samples; lanes "4–6" and "10–12": proteins from drought treated leaves. Red arrow points to a high abundant protein band which decreased in intensity in samples treated with ProteoMiner; Green arrow points to a protein band of which the intensity was increased after the ProteoMiner enrichment process.

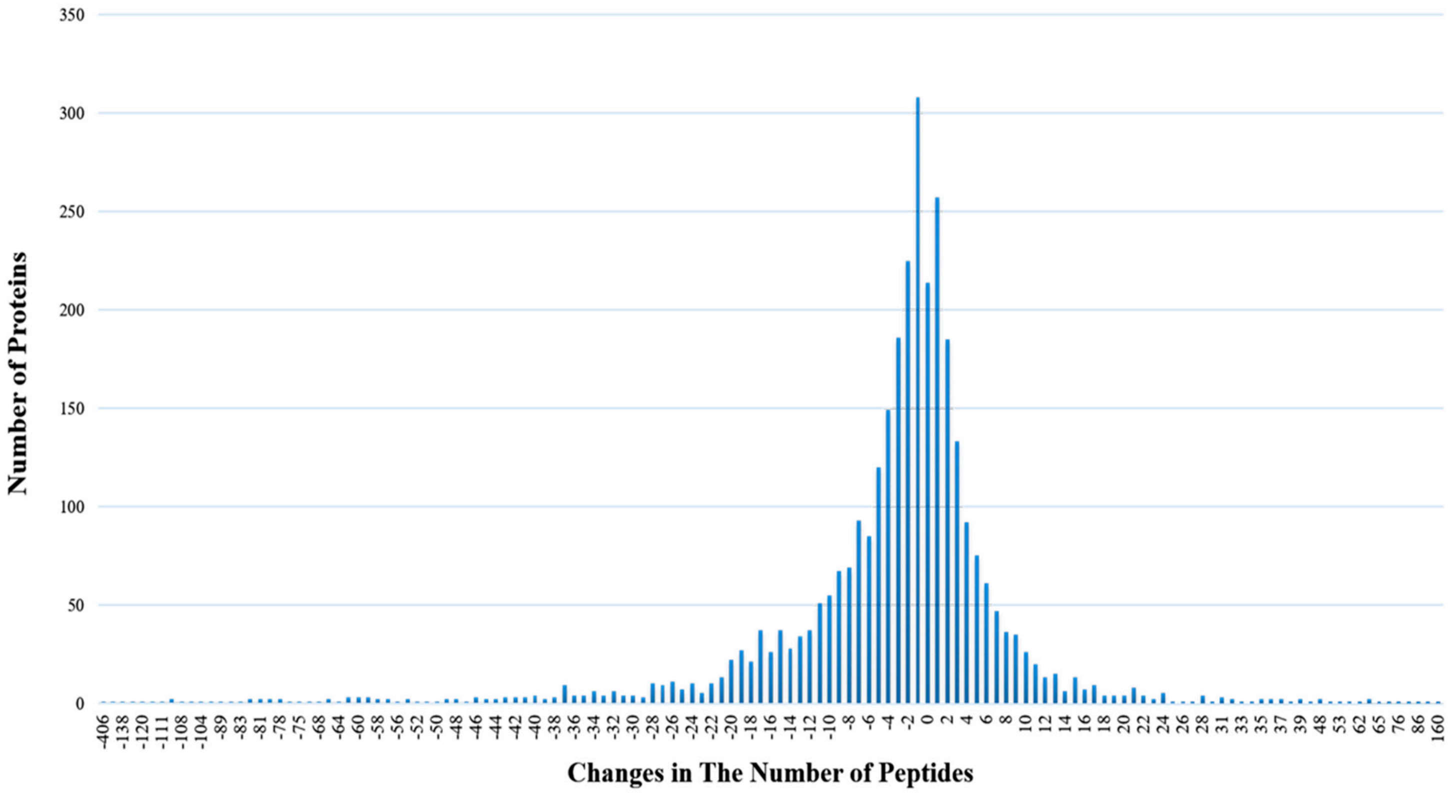

Figure S2. The effect of ProteoMiner enrichment on the number of peptides identified to individual proteins in switchgrass leaf proteomes. This figure shows the changes in the number of peptides identified in individual proteins after the ProteoMiner-enrichment procedure. The ProteoMiner-enrichment showed no effects on 214 proteins which were identified with the same number of peptides with or without the procedure. For the remaining proteomes, the Proteominer enrichment procedure resulted in the identification of either more peptides (1–160) or less (−1 to −406) peptides in each individual protein. For the majority of these proteins, the differences in the number of peptides identified to one protein are in the range from −28 to 20 peptides between samples with or without the Proteominer enrichment procedure.

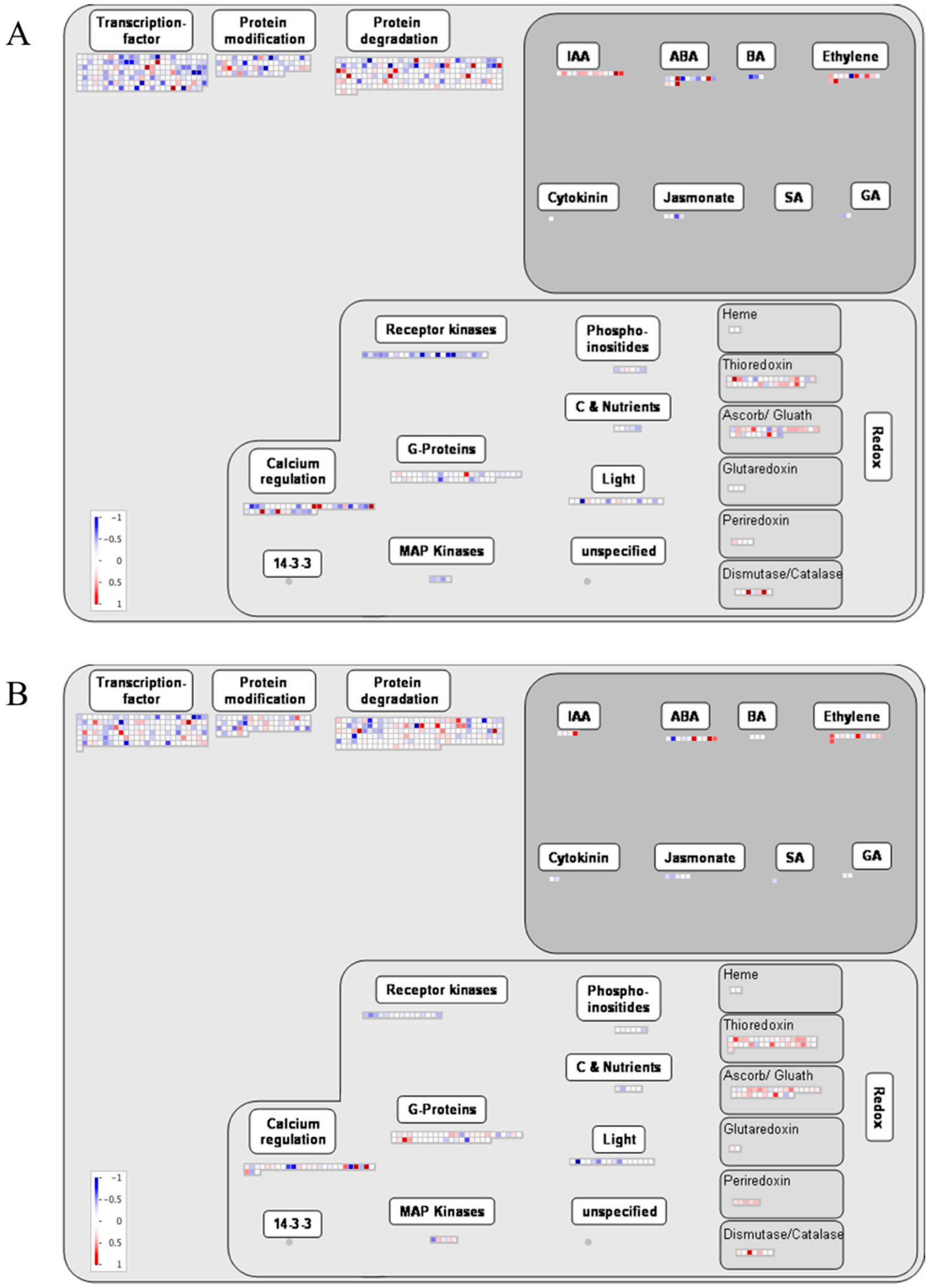

Figure S3. Molecular function overview of leaf proteomes quantified in either the crude leaf protein extracts or the ProteoMiner enriched protein samples from drought-treated swticghrass. The graph was generated using the Regulation_Overview and Ath_AGI_ISOFORM_MODEL_TAIR10_Aug2012 as the reference database in MapMan. (**A**) molecular function overview of proteomes identfied in the crude leaf protein extracts; (**B**) molecular function overview of proteomes identfied in the ProteoMiner-enriched samples. The intensity of the color change corresponds to the scale created based on log2 fold of protein from drought-treated to untreated groups. The relevant annotated accession numbers of identified proteins associated with *Arabidopsis thaliana* database were used to categorize proteins into different cell functional groups by MapMan.
